# Identification of Putative Rhamnogalacturonan-II Specific Glycosyltransferases in Arabidopsis Using a Combination of Bioinformatics Approaches

**DOI:** 10.1371/journal.pone.0051129

**Published:** 2012-12-14

**Authors:** Aline Voxeur, Aurélie André, Christelle Breton, Patrice Lerouge

**Affiliations:** 1 Laboratoire Glyco-MEV, EA 4358, Institut de Recherche et d'Innovation Biotechnologique, University of Rouen, Mont-Saint-Aignan, France; 2 CERMAV-CNRS, University of Grenoble 1, Grenoble, France; Ghent University, Belgium

## Abstract

Rhamnogalacturonan-II (RG-II) is a complex plant cell wall polysaccharide that is composed of an α(1,4)-linked homogalacturonan backbone substituted with four side chains. It exists in the cell wall in the form of a dimer that is cross-linked by a borate di-ester. Despite its highly complex structure, RG-II is evolutionarily conserved in the plant kingdom suggesting that this polymer has fundamental functions in the primary wall organisation. In this study, we have set up a bioinformatics strategy aimed at identifying putative glycosyltransferases (GTs) involved in RG-II biosynthesis. This strategy is based on the selection of candidate genes encoding type II membrane proteins that are tightly coexpressed in both rice and Arabidopsis with previously characterised genes encoding enzymes involved in the synthesis of RG-II and exhibiting an up-regulation upon isoxaben treatment. This study results in the final selection of 26 putative Arabidopsis GTs, including 10 sequences already classified in the CAZy database. Among these CAZy sequences, the screening protocol allowed the selection of α-galacturonosyltransferases involved in the synthesis of α4-GalA oligogalacturonides present in both homogalacturonans and RG-II, and two sialyltransferase-like sequences previously proposed to be involved in the transfer of Kdo and/or Dha on the pectic backbone of RG-II. In addition, 16 non-CAZy GT sequences were retrieved in the present study. Four of them exhibited a GT-A fold. The remaining sequences harbored a GT-B like fold and a fucosyltransferase signature. Based on homologies with glycosyltransferases of known functions, putative roles in the RG-II biosynthesis are proposed for some GT candidates.

## Introduction

**Figure 1:**
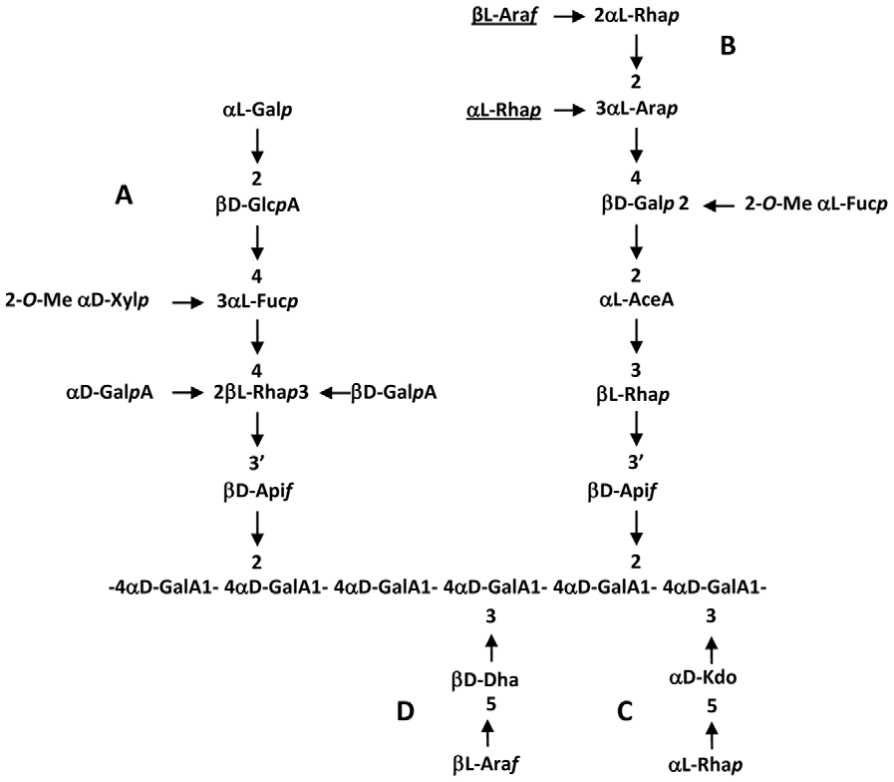
The glycosyl sequence of RG-II. RG-II is composed of an α-1,4-linked homogalacturonan backbone that is substituted with four side chains A to D. The underlined sugars are absent in Arabidopsis. Only the apiosyl residue of side chain A is involved in the borate-diester cross-linking of RG-II.

Pectins are complex acidic polysaccharides of the primary cell wall containing three distinct domains: homogalacturonan (HA), rhamnogalacturonan-I (RG-I) and rhamnogalacturonan-II (RG-II). RG-II is the most structurally complex pectic polysaccharide and is composed of an α(1,4)-linked homogalacturonan backbone substituted with four structurally different oligosaccharide side chains A to D [1] (Fig. 1). At least twelve different glycosyl residues are present in RG-II, including 3-deoxy-d-*manno*-octulosonic acid (Kdo) and the rare aceric acid (AceA), apiose (Api), and 3-deoxy-d-*lyxo*-heptulosonic acid (Dha) [2],[3]. In addition, L-arabinose exists in both pyranose and furanose forms and the hexose residue located at the non-reducing end of the side chain A, originally reported as a D-galactose, has been shown to be in the L-configuration [4],[5]. Despite its highly complex structure, RG-II is evolutionarily conserved in the plant kingdom as it is present in the primary cell wall of all higher plants predominantly in the form of a dimer that is cross-linked by a borate di-ester between two apiosyl residues of side chain A [2],[6].This suggests that proteins involved in its synthesis appeared early in land plant evolution and that RG-II has fundamental functions in the primary wall organisation. So far, all mutations affecting the RG-II structure and in muro dimerization significantly alter the plant development [5],[7],[8] Although RG-II is believed to play a key function in primary cell wall formation, its biosynthesis is poorly understood. To date, mainly the biosynthesis of RG-II specific nucleotide sugars has been elucidated [8],[9],[10]. With regards to glycosyltransferases (GTs) involved in RG-II biosynthesis, only a unique xylosyltransferase activity has been so far biochemically characterised out of approximately 20 GTs that are required for the synthesis of this pectic molecule. This α3-xylosyltransferase (α3-XylT), named RGXT, is able to transfer a xylose residue onto the fucose of the side chain A [11]. The Arabidopsis RGXT family has four members, but RGXT4 was the only member to be clearly linked to RG-II synthesis [12]. The functional characterization of RXGT4, encoded by the *MALE GAMETOPHYTE DEFECTIVE* 4 (*MGP4*) gene, has demonstrated that the mutation in this gene affects both the structural integrity of RG-II and the normal growth of roots and pollen tubes in Arabidopsis [12].

The study of mutants affected in the RG-II biosynthesis has shown that RG-II alteration results in either strong developmental phenotypes or lethality [8],[9],[10],[12]. As a consequence, the identification of new RG-II GT candidates through a conventional reverse genetic strategy is unlikely to be feasible. Furthermore, since RG-II is composed of rare monomers or monomers exhibiting unusual ring forms or configurations, it is tricky to identify putative GT candidates from a search of sequence homologies with well-characterised GTs from other organisms that are listed in the Carbohydrate-Active enZyme (CAZy) database (http://www.cazy.org/). Attempts to identify non-CAZy GTs through bioinformatics approaches have been previously carried out irrespectively of a target cell wall polysaccharide. These strategies aimed to select in the Arabidopsis genome new GT candidates exhibiting a type II membrane topology typical of Golgi-resident proteins and GT signatures [13],[14]. Recently, co-expression analysis has emerged as a powerful way to identify functionally related genes to query genes. Gene expression patterns by microarray experiments provide information about candidate proteins involved in the same biological process. Coexpression analyses can be carried out using web-based bioinformatics tools, such as the ATTED-II, GeneCAT or PlaNet co-expression databases [15],[16],[17]. In the present study, we report on the setting up of a bioinformatics scheme designed for the specific identification of putative GTs involved in the biosynthesis of RG-II. This strategy is mainly based on the search in plant genomes of genes coexpressed with sequences encoding well-characterised enzymes involved in either the cytosolic synthesis of RG-II specific monomers or in its Golgi biosynthesis.

## Materials and Methods

### Co-expression analysis

Co-expression information for *Arabidopsis thaliana* and *Oryza sativa* was obtained from the ATTED-II (http://atted.jp) [15]. Source of GeneChip data in ATTED-II version 5.5 are for *A. thaliana*, the 1388 Affymetrix (ATH1) arrays from the 58 experiments on each developing stage, biotic and abiotic treatment obtained from TAIR (http://www.arabidopsis.org/) and for rice, the 310 arrays obtained from ArrayExpress [18]. Since ATTED-II website provides only the top 300 genes co-expressed with query gene, we downloaded co-expressed gene tables from http://atted.jp/top_download.shtml.

### Genomic Resources

For *Arabidopsis thaliana* and *Oryza sativa* genome analysis, we retrieved protein sequences respectively from TAIR (www.arabidopsis.org) and RAP-DB (http://rapdb.dna.affrc.go.jp/) websites. Correspondences between RAP ID and TIGR ID were obtained thanks to PlantArrayNet [19]. For phylogenetic analysis of GT families, protein sequences were retrieved from NCBI (http://www.ncbi.nlm.nih.gov/) and Plaza database (http://bioinformatics.psb.ugent.be/plaza/) [20].

### Transmembrane helix prediction

Predictions of transmembrane helices were carried out using the TMHMM server version 2.0 [21] (http://www.cbs.dtu.dk/services/TMHMM). All predictions were performed using standard settings. Proteins predicted to contain at least one transmembrane domain (TMD) within the N-terminal first 150 amino acid residues were selected. ARAMEMNON consensus prediction was used for confirmation [22].

### Phylogenetic profile

Phylogenetic profile for each candidate gene has been retrieved from OrthoMCL database [23], Plaza comparative genomics platform [24] and Phytozome [25].

### Isoxaben data

Microarray data were obtained from the Nottingham Arabidopsis Stock Centre (NASCArrays Experiment Reference Number: NASCARRAYS-27; http://affymetrix.arabidopsis.info/narrays/experimentbrowse.pl). Data covering 3 control experiments and 3 experiments after treatment with isoxaben were analyzed with respect to changes in expression level (spot signal) of each gene. The significance of difference between control and isoxaben was estimated by carrying out a one-tailed paired t-test.

### Phylogenetic analysis

Full length protein sequences were aligned using ClustalW [26] with the PAM protein weight matrix, pairwise gap opening/extension penalties of 10/0.1, and multiple alignment gap opening/extension penalties of 10/0.2. Phylograms were constructed from the aligned sequences using the neighbour-joining method [27]. The tree is drawn to scale with branch length in the same units as those of the evolutionary distances used to infer the phylogenetic tree. The evolutionary distances were computed using the Poisson correction method [28] and scale bar represents number of amino acid substitution per site. Phylogenetic tree analyses were conducted in MEGA 4 [29].

### Coexpression network

The edge force directed coexpression networks for rice and Arabidopsis were generated with Cytoscape 2.8 (http://www.cytoscape.org) from data retrieved from ATTED-II [15]. An intersection coexpression network was then generated from the two networks using the Cytoscape Merge Network plug-in.

### Fold recognition analysis and Hydrophobic Cluster Analysis method (HCA)

The protein sequences of non-CAZy candidates were submitted to a fold recognition analysis using the PHYRE Web server (http://www.sbg.bio.ic.ac.uk/phyre2/html/page.cgi?id=index), a fully automatic programme that performs a profile-profile matching algorithm together with predicted secondary structure matching (http://www.sbg.bio.ic.ac.uk/phyre2/html/page.cgi?id=index) [30]. Sequences were submitted in the normal mode, and those giving a GT fold in the top ten hits, particularly with a high or moderate confidence level (typically above 85%) were retained. The relevant candidates from this analysis were then submitted to the Hydrophobic Cluster Analysis method (HCA). HCA is a graphical method based on the detection and comparison of hydrophobic clusters that are presumed to correspond to the regular secondary structure elements constituting the architecture of globular proteins [31],[32]. For the trained user, HCA is a powerful method to detect conserved structural motifs in highly divergent sequences (typically less than 20% of sequence identity). HCA plots were obtained from: http://bioserv.impmc.jussieu.fr/hca-form.html.

## Results

### Selection of GT candidates

**Figure 2:**
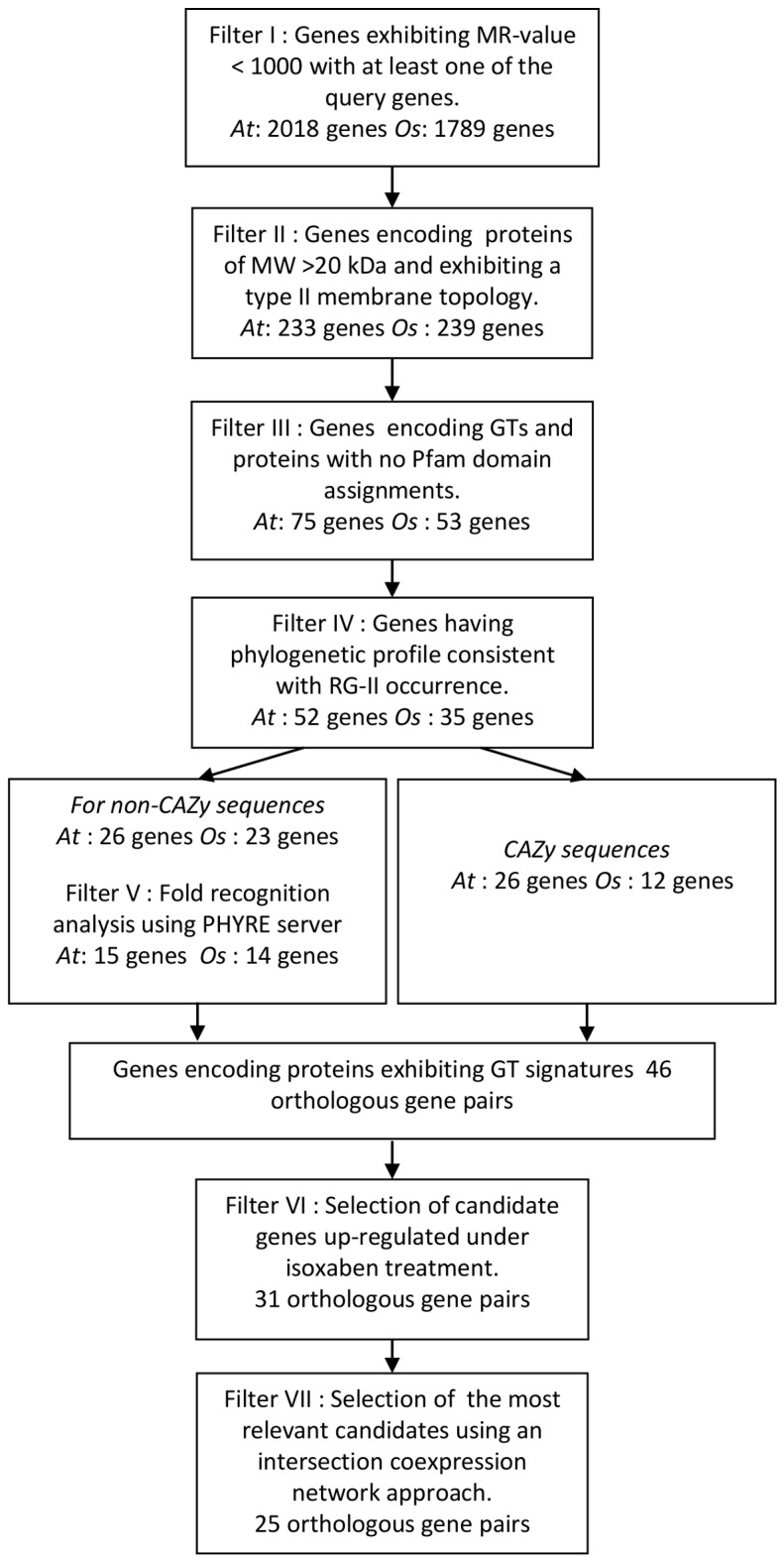
Flow chart of the 7-step filtering strategy used to select putative RG-II specific GTs. At : *Arabidopsis thaliana*, Os : *Oryza sativa*.

In an effort to select GTs potentially involved in RG-II biosynthesis, we adopted a bioinformatics approach based on the following filtering process (Fig. 2):

#### Filter I: Coexpression with genes encoding RG-II related enzymes

To identify candidate genes involved in RG-II biosynthesis, we used the CoexSearch tool available at ATTED-II. This gene coexpression database uses mutual rank (MR) value as coexpression measure and provides a comparative view between Arabidopis and rice coexpression using orthologous genes. A low MR value (below 1,000) was demonstrated to be appropriate to highlight tightly coexpressed genes involved in a same biological process [15],[33], including the biosynthesis of cell wall polymers [34]. Coexpression analyses require an appropriate selection of query genes (guide genes). As a consequence, we used as queries only genes specifically involved in RG-II biosynthesis. *RGXT* genes, encoding α3-XylTs that are to date the only well-characterised GT activities involved in RG-II biosynthesis, were considered. In rice and Arabidopsis genomes, one and four homologues were identified, respectively. Among the four Arabidopsis homologues, *AtRGXT1-2* exhibit too low expression levels for accurate co-expression studies and no expression data were available for *AtRGXT3*. In contrast, *AtRGXT4* (At4g01220) exhibits a higher expression level in plant tissues [35] and encodes an isoform of the XylT that was demonstrated to be required for normal plant growth [12]. As a consequence, *AtRGXT4* and the rice homologue *OsRGXT* (Os05g32120) were selected as guide genes (Table 1).

**Table 1:** List of genes encoding putative glycosyltransferases involved in RG-II biosynthesis that were selected in *Arabidopsis thaliana* and *Oryza sativa* genomes through the bioinformatics filtering process.

				Guide genes					Guide genes		
	Name	Isoxaben up-regulation	p-value	MR *AtKDSA1*	MR *AtKDSA2*	MR *AtRGXT4*	MR *AtKDSB*	Rice orthologues	MR *OsKDSA2*	MR *OsRGXT*	MR *OsAXS*
**CAZy GT**											
**GT4**											
At1g19710		49%	0.06	2287	**301**	**472**	1350	Os01g15780	n.a.	n.a.	n.a.
**GT8**											
At5g47780	AtGAUT4	80,30%	0.05	1802	1152	**229**	1225	Os09g30280	1024	3374	**312**
At2g38650	AtGAUT7	44,70%	0.06	**446**	**428**	**68**	2745	Os07g48370	**157**	**389**	**46**
At3g25140	AtGAUT8	118,60%	<0,01	1724	1914	**16**	1767	Os02g29530	3444	3101	**424**
At3g61130	AtGAUT1	40,1%	0.03	**668**	2655	**6**	3867	Os06g49810	**430**	**405**	**1**
**GT29**											
At1g08660		49,80%	<0.01	**6**	**16**	**619**	**216**	Os11g36420	6688	2167	2767
At3g48820		n.a.	n.a.	n.a	n.a.	n.a.	n.a.	Os02g02620	**93**	**277**	**626**
**GT31**											
At5g53340		21.8%	0.06	1622	1025	**345**	4781	Os08g29710	4324	1613	1389
**GT68**											
At5g50420		117,90%	<0.01	**168**	**152**	**175**	1511	Os07g38490	**480**	**227**	**151**
**GT92**											
At2g33570		55%	0.01	5180	3845	**165**	**675**	Os06g22330	**523**	1162	**266**
**Non CAZy GT**											
**GT-A like**											
**No PFAM**											
At5g12260		44%	0.02	1359	1984	**900**	3047	Os01g18060	**1035**	**19**	**839**
**DUF616**											
At4g38500		21,90%	0.04	7180	7086	2147	5157	Os06g50860	**739**	**114**	3909
**DUF707**											
At1g61240		106,30%	0.04	3611	3980	15503	**408**	Os06g51520	**245**	**340**	**80**
At2g28310		338%	<0.01	**134**	**17**	1724	**542**	Os02g19510	9583	2843	4018
**GT-B like**											
**PF10250**											
At1g04910		20,60%	<0.01	4776	4731	**88**	1120	Os11g29120	6740	**813**	8695
								Os12g23760	**209**	**114**	**640**
At1g14020		32,90%	<0.01	7411	9965	1377	11503	Os03g07310	1819	**154**	2059
At2g03280		76,30%	0.02	8093	4390	11931	1136				
At4g16650		57%	0.05	1722	1312	5379	**752**	Os04g46570	n.a.	n.a.	n.a.
At1g62330		61,10%	0.05	**83**	**43**	**722**	**497**	Os06g13215.	**394**	**747**	1470
At3g26370		20,30%	0.01	2256	**680**	1402	2183	Os02g06400	**544**	**169**	**294**
								Os06g47290	**580**	**81**	3636
At3g30300		41%	0.04	**225**	**62**	2208	1316	Os05g04190	3336	**172**	2088
At3g21190		51%	0.07	1412	**531**	**304**	**805**	Os09g32320	**29**	1295	**69**
**Other**											
At3g26950		33%	0.02	5971	3354	1496	**126**	Os11g31110	2496	1231	3570
At3g56750		34%	0.09	1587	**886**	3785	**72**	Os07g44700	**366**	1582	**67**
At4g12700	SUL1	109,60%	0.01	4766	3319	7609	3955	Os10g31810	**186**	**18**	**377**
At4g08810	SUB1	n.a.	n.a.	n.a	n.a.	n.a.	n.a	Os07g43990	**838**	2512	**66**

n.a.: no transcriptomic data available. MR values in bold are representative of tightly co-expressed genes.

Five specific monomers are required for RG-II biosynthesis: L-Gal, Dha, AceA, Api and Kdo. No information is available so far about Dha and AceA biosynthesis. The biosynthesis of GDP-L-Gal is not specifically related to the RG-II biosynthesis because this nucleotide-sugar is also involved in the synthesis of ascorbate [5],[36]. In contrast, Kdo is specifically dedicated to the Golgi biosynthesis of RG-II [10]. This ketoacid is synthesized in the cytosol *via* the action of Kdo-8-P synthase (KDSA), catalysing the condensation of phosphoenolpyruvate onto d-Ara-5-P, and CMP-Kdo synthetase (KDSB) involved in the activation of Kdo as a nucleotide sugar. Two *KDSA* genes (At1g79500/*AtKDSA1* and At1g16340/*AtKDSA2*), exhibiting similar expression levels and profiles [10], and one *KDSB* gene (At1g53000/*AtKDSB*) [37] were identified in *A. thaliana*. In the *Oryza sativa* genome, two *KDSA* orthologues (Os07g28690/*OsKDSA1*, Os12g10784/*OsKDSA2*) and one *KDSB* (Os05g48750/*OsKDSB*) orthologue were predicted. No transcriptomic data were available for *OsKDSA1* and as a consequence only *AtKDSA1-2, AtKDSB*, *OsKDSA2 and OsKDSB* were selected. Finally, genes encoding UDP-D-apiose/UDP-D-xylose synthase (AXS) involved in UDP-Api biosynthesis were also used as guide genes [8],[38]. Data regarding the expression of *AXS* in Arabidopsis being lacking, only the rice orthologue was selected (Os01g73790/*OsAXS*). In order to make sure that the selection of guide genes was well performed, we checked that they exhibited similar expression patterns confirming their involvement in a same biological process. Only one rice gene, *OsKDSB*, displaying very high MRs with other rice guide genes (>10,000), was excluded from our query gene list (data not shown).

ATTED-II co-expression database was queried with each guide gene and sequences exhibiting low coexpression MR values (MR<1,000) [33] with at least one of Arabidopsis or *Oryza sativa* query genes were selected as putative candidates. This led to the selection of 2018 and 1789 genes for *A. thaliana* and *O. sativa*, respectively (Fig. 2).

#### Filter II: Genes encoding type II proteins of MW>20 kDa

Among these candidates, we only conserved genes encoding proteins of MW>20 kDa and exhibiting a type II membrane topology predicted by TMHMM2.0 server [21]. ARAMEMNON consensus prediction was used for confirmation [22]. This filtering step led to 233 sequences from *A. thaliana* and 239 sequences from *O. sativa*.

#### Filter III: Selection of GT or proteins with unknown function

Among the retrieved candidates using CoexSearch tool, we first conserved those belonging to CAZy GT families. For other genes, we submitted the corresponding protein sequence for Pfam matches (pfam.sanger.ac.uk). Most of selected sequences exhibited oxydase, hydrolase or methyltransferase domain. We selected only genes encoding proteins with GT Pfam, with domain of unknown function (DUF) or encoding proteins with no predicted domain. This results in a list of 75 *A. thaliana* and 53 *O. sativa* genes.

#### Filter IV: Phylogenetic profiling

Phylogenetic profiling is based on the concept that functionally related genes are gained and lost together from genomes during evolution [39]. *AtRGXT4*, which encodes the unique RG-II GT reported so far, displays orthologues in all land plants whose genomes have been sequenced up to now and not in other eukaryote and prokaryote genomes. This is consistent with the reported occurrence of RG-II [1]. In contrast, since Kdo and Api are not plant specific sugars [40], [41], the genes involved in their biosynthesis do not have a phylogenetic profile strictly corresponding to the RGII occurrence. Consequently, the only guide gene used for this analysis was *AtRGXT4*. As for this gene, all genes encoding GT involved in RG-II biosynthesis should also exhibit orthologues specifically in land plant genomes. All candidate genes previously selected and sharing phylogenetic profile similar to the one of *AtRGXT4* were considered as coding putative RG-II specific GTs. As a first step, OrthoMCL database [23] was used in order to select genes not having more than one orthologue in a clade different from the Viridiplantae. As a second step, thanks to the Plaza comparative genomics platform [24] and Phytozome [25], we selected among these Viridiplantae specific genes those without orthologues in Chlorophytes sequenced to date and presenting at least one orthologue in each Angiosperm sequenced species. The lack of orthologues in *Physcomitrella patens* and/or *Selaginella moellendorffii* genomes was not considered since their RGII structures have not been characterised yet. This phylogenetic profiling step resulted in the selection of 52 *A. thaliana* and 35 *O. sativa* genes. Genes that have been excluded at this step are listed in Table S1 for information purpose.

#### Filter V: Fold recognition analysis

At the structural level, all of the nucleotide-sugar-dependent GTs solved to date have revealed only two structural folds called GT-A and GT-B [42],[43]. Therefore the use of fold recognition methods appears to be appropriate for this class of enzymes to tentatively identify a GT signature in the protein sequences that have been sorted out in the present study. Among the previously selected candidates, 26 Arabidopsis and 12 from *O. sativa* are already listed in the CAZy database. We submitted to the fold recognition program PHYRE all of the remaining retrieved non-CAZy protein sequences [30]. When the results of scanning a query sequence against a library of known protein structures revealed a GT as structural homolog with a high or moderate score (confidence level >85%), the corresponding gene was selected as encoding a putative RG-II specific GTs. Among the 26 *A. thaliana* and the 23 rice sequences submitted to PHYRE, 15 and 14 sequences were retained as putative GT, respectively.

The comparison of the lists of candidate genes, having expression data in both *A. thaliana* and *O. sativa*, indicated that the five-step filtering strategy results in the selection of orthologous candidate genes in both species. Thus, the 41 *A. thaliana* and 26 *O. Sativa* candidates corresponded to 40 and 24 orthologous gene families, respectively, of which 18 common to the two plants. The two gene lists were combined into a single list composed of 46 orthologous gene families. For some candidate gene families, either Arabidopsis or rice genes exhibit high MRs (*i.e.* At1g14020, At1g61240, At4g38500, Os02g29530, Os09g30280 and Os11g36420 in Table 1) and were selected because their orthologues exhibited strong correlations with their relative guide genes. The reported expression levels of these genes are low and as a consequence less suitable for coexpression analysis. This could explain this discrepancy between Arabidopsis and rice orthologous genes of the same family [15].

#### Filter VI: Selection of candidate genes up-regulated under isoxaben treatment

The herbicide isoxaben inhibits cellulose biosynthesis and isoxaben-habituated plants compensate the loss of cellulose-xyloglucan load bearing network by constructing walls predominantly made of pectin [44]. Analysis of microarray data obtained from isoxaben-habituated Arabidopsis cells showed that *AtKDSA1, AtKDSA2*, *AtKDSB* and *AtRGXT4* are significantly up-regulated (1.3 to 1.6 fold) upon treatment with this herbicide (http://affymetrix.arabidopsis.info/narrays/experimentbrowse.pl). Among the 46 retrieved Arabidopsis sequences, 31 candidate genes were also found to exhibit an up-regulation of at least 20% in these conditions. Because isoxaben mainly induces the accumulation of HA domain in the plant cell wall [44] and considering that Arabidopsis guide genes are overexpressed in this condition and that RG-II is composed of HA substituted by four side-chains, we postulate that these 31 candidate genes, overexpressed under isoxaben treatment, potentially encode RG-II GTs.

Among genes that are not overexpressed upon isoxaben treatment and as a consequence not selected as candidate genes in this study, it should be mentioned *ARAD1* that encodes a putative arabinosyltransferase involved in the biosynthesis of RG-I [45], *FUT1, XXT5* and *MUR3* encoding xyloglucan-specific GTs (Table S2). In contrast, XXT2 (At4g02500), a GT34 xylosyltransferase also involved in xyloglucan biosynthesis [46] was initially selected as a putative candidate in the filtering strategy. Although the overexpression (225%) of *XXT2* upon isoxaben treatment is questionable, this sequence was no longer considered because the expression level of this gene was too low to be relevant.

#### Filter VII: coexpression networks

**Figure 3:**
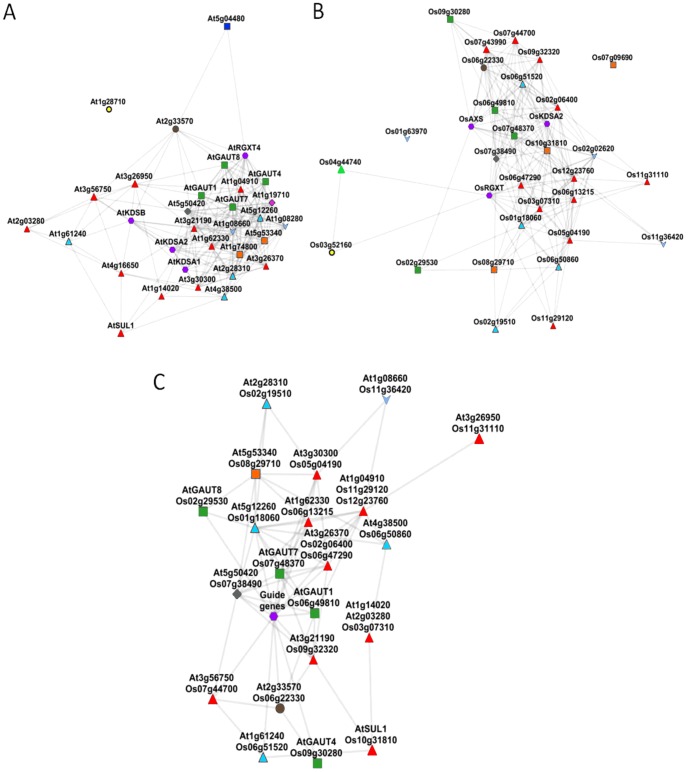
Coexpression and intersection coexpression networks. Gene coexpression networks for (A) Arabidopsis and (B) *Oryza sativa* GT candidate genes generated using an edge weighted force directed approach, based on data retrieved from ATTED-II and visualised in Cytoscape 2.8 (http://www.cytoscape.org). C) Intersection coexpression generated from the two networks in order to highlight conserved coexpression relationships between networks of the two species. • (purple): Guide genes (*AtRGXT4, AtKDSA1-2, AtKDSB, OsRGXT, OsKDSA2, OsAXS*), ⧫ (pink): GT4, ▪ (green): GT8, ▴ (blue): GT29, ▪ (orange): GT31, ▴(green): GT32, ⧫ (grey): GT68, • (brown): GT92, ▴ (blue): non-CAZy GT-A like, ▴ (red): non-CAZy GT-B like, ○ (yellow): GT77, ▪ (blue): GT1-like.

In a final attempt to have a global insight of coexpression relationships linking candidate genes and to exclude the less relevant orthologous gene families, we constructed a gene coexpression network for each species. We used an edge weighted force directed approach, based on data retrieved from ATTED-II and visualised in Cytoscape 2.8 (http://www.cytoscape.org). In these networks, each gene is represented by a node and each edge connecting two nodes represents an MR<1000. In each network, module of high density was revealed suggesting a tight coexpression (Fig. 3A and B). An intersection coexpression network was then generated from the two networks using the Cytoscape Merge Network plugin in order to highlight conserved coexpression relationships between networks of the two species (Fig. 3C). Guide gene nodes were collapsed in a unique node and for candidate genes we used orthologous gene relationship resulting in a one-to-one or two-to-one mapping between the nodes of the two networks. Thus, the *Arabidopsis thaliana-Oryza sativa* intersection network is defined as the network over the set of nodes where there is a link between two nodes i and j if i and j denote two pairs of orthologous genes which are connected in both Arabidopsis and *Oryza sativa* networks.

The coexpression networks revealed that some candidate genes selected on the basis of their MR value with one of the guide genes exhibit none conserved coexpression with either other candidates or guide genes and are consequently absent from the intersection network. In contrast, 21 orthologous gene families exhibit strong conserved coexpression patterns. Four other genes (At4g16650, At1g19710, Os02g02620 and Os07g43990), having expression data in only one of the two species and exhibiting strong coexpression relationship with other candidate genes either in Arabidopsis or *Oryza sativa*, were retained as additional putative RG-II GT candidates. Table 1 gathers the RG-II GT candidates selected throughout the bioinformatics 7-step filtering process. Sequence information about these putative GTs is provided in Table S3. As expected, most GT candidates have been identified in the Arabidopsis Golgi proteome (Table S4) [47].

### Evaluation of the candidate genes

#### CAZY GT candidates

In order to identify GTs involved in RG-II biosynthesis, 10 candidates listed in the CAZy database were selected in the 7-step filtering process. They belong to families GT4, 8, 29, 31, 68, and 92.

#### GT8

Within the large family GT8, the Arabidopsis GAUT family comprises 15 protein members. The selection of several GAUT members (GAUT1, GAUT4, GAUT7 and GAUT8) is a good indicator of the filtering efficiency because two of these transferases, GAUT1 and GAUT7, have been demonstrated to be associated in the Golgi apparatus to form the galacturonosyltransferase complex responsible for the synthesis of α4-GalA oligogalacturonides constitutive of both homogalacturonan and RG-II backbones [48],[49]. Another *GAUT* gene (*GAUT8/QUA1*) has been suggested to encode a transferase involved in pectin synthesis based on the phenotypes of plant lines carrying mutations in this gene [50]. In contrast, another GAUT member, GAUT12/IRX8, has been proposed to be an α4-GalAT involved in the synthesis of the reducing end of xylan [51],[52]. Present data strengthen previous observations that GAUT members are involved in pectin synthesis [49],[53],[54],[55] and more particularly highlights a possible participation of GAUT1, 4, 7 and 8 in RG-II synthesis, as the corresponding genes are tightly co-expressed with *AtRGXT4* (Table 1). Although GAUT4 exhibits high homology with GAUT1, 7 and 8 involved in the synthesis of the oligogalacturonide backbone of pectins, we cannot rule out that this α-GalAT candidate is responsible for the transfer of GalA onto the rhamnose residue of the side chain A.

#### GT29

In addition to the GAUT sequences, two sialyltransferase-like sequences (ST-like), At1g08660, At3g48820, were also selected. In contrast to the third Arabidopsis sequence At1g08280 listed in family GT29, these two proteins were previously reported to present the four conserved sialyltransferase motifs [56] and were shown to be located in the Golgi apparatus [47]. Since sialic acids are absent in plants [57] and considering that sialic acid and Kdo transferases share common features such as the use of CMP-activated nucleotide sugars as substrates, we have previously proposed that At1g08660 and At3g48820 could be involved in the transfer of Kdo and/or Dha on the pectic backbone of RG-II [58]. Moreover, mutation of At1g08660 was demonstrated to induce defects in pollen germination and pollen tube growth [59] as reported for *Atkdsa1/Atkdsa2* mutants impaired in Kdo synthesis [10].

#### GT4

One of the candidates, At1g19710, is listed in the very large family GT4 which contains sequences encoding retaining transferases harboring a GT-B fold. Plant accessions reported in family GT4 are involved in a wide range of biological functions including sucrose metabolism, synthesis of chloroplast galactolipids and of the GPI anchor. In contrast to most plant GT4 members, protein encoded by At1g19710 gene exhibits a type-II topology and has previously been detected in the Golgi apparatus [47] suggesting a potential role in non-cellulosic polysaccharide biosynthesis. At1g19710 is tightly coexpressed with *GAUT* and GT29 genes as shown in Fig. 3A. Despite sequence similarities with other GT4 accessions, At1g19710 and its rice orthologue represent a distinct lineage as seen in the phylogenetic tree depicted in Fig. S1, but no indication of its precise biochemical function could be anticipated for this sequence.

#### GT31

Members of family GT31 encode mainly inverting ß3-glycosyltransferases (ß3-GalTs, ß3-GalNAcTs, ß3-GlcTs, etc). This family is one of the most populated families with plant sequences (33 Arabidopsis sequences) but only one plant sequence (a ß3-GalT, also called GalT1) has been characterized to date. This protein, GalT1, is involved in the synthesis of Lewis a epitopes in *N*-glycans [60]. One member of GT31 was selected in this study, At5g53340, and it belongs to clade 10 as reported in Egelund et al. [61]. It must be stressed that At5g53340 is also tightly coexpressed with *GAUT7*, GT4 and GT29 genes as shown in the coexpressed gene networks provided by ATTED-II. On the basis of what is known for members of GT31 family, one can speculate for At5g53340 the possible formation of a ß3-glycosidic linkage (from an α-linked nucleotide sugar) as the one observed in RG-II side chain A (GalAß1-3)Rha).

#### GT68

The family GT68 contains protein accessions sharing sequence identities with protein *O*-fucosyltransferases (POFUT2) characterized in Drosophila and human [62], [63]. These enzymes transfer an α-l-fucose residue from GDP-fucose to a conserved serine or threonine residue in thrombospondin type 1 repeats (TSRs). Protein-*O*-fucosylation is an unusual form of glycosylation that has only been observed in Epidermal Growth Factor (EGF)-like repeats and TSR repeats. These small protein motifs are found in hundreds of cell-surface and secreted proteins in metazoans and their modification by *O*-fucose is believed to modulate signal transduction pathways. Three *A. thaliana* genes encoding proteins of unknown function are listed in family GT68. Among them, At5g50420 and its rice ortholog Os07g38490 exhibited strong coexpression patterns with guide genes and *GAUT1-7* in both *A. thaliana* and *Oryza sativa* (Table 1, Fig. 3). Phylogenetic tree (Fig. S2) shows that plant GT68 sequences are contained in a distinct clade from other accessions of the family.

The link between these plant sequences and RG-II biosynthesis is not straightforward, except if we consider a more general function as a fucosyltransferase, or alternatively as a possible way to modulate cell wall biosynthesis through specific *O*-fucose modification of regulatory proteins. It must also be stressed that members of family GT68 belongs to the large fucosyltransferase superfamily that has been previously described [64] and which includes protein sequences from four other CAZy GT families (GT11, GT23, GT37, GT65). This superfamily is characterized by the presence of three conserved peptide regions (see paragraph on GT-B-like sequences).

#### GT92

CAZy family GT92 contains inverting enzymes from various organisms except mammals, exhibiting a DUF23 domain. In this family, two ß4-galactosyltransferases involved in the synthesis of unique *N*-glycan structures, such as the core Galß(1–4)Fuc epitope found in proteins of the nematode *C. elegans*, have been characterized [65],[66]. Plant GT92 proteins, including three Arabidopsis sequences, are contained in a distinct clade from other proteins (Fig. S3). At2g33570 is one of the candidate genes that have been selected in this study. In addition, this gene appears to have a clear link with cell wall biosynthesis since it is also tightly coexpressed with *CesA6* encoding a cellulose synthase and cell wall proteins (*i.e.*, extensin, AGP, etc). If we assume a similar function (ß4-linkage forming enzyme) for this sequence, an hypothetical role for this protein sequence could be the addition of a ß(1–4)GlcA to the fucose residue of RG-II side chain A.

#### Non-CAZy GT candidates

The combined use of the fold recognition programme PHYRE [30] and the Hydrophobic Cluster Analysis method (HCA) [31],[32] was an efficient approach to identify possible GT signatures in the protein sequences of unknown function that were retrieved using the present bioinformatics strategy (non-CAZy sequences listed in Table 1). Among the 16 retrieved non-CAZy candidate protein sequences, five gave strong scores using PHYRE with GTs known to adopt a GT-A fold and the remaining sequences are predicted to adopt a GT-B fold.

#### GT-A like sequences

The GT-A fold type is shared by numerous GT families (currently 18) in CAZy, including inverting and retaining families. A similar fold type is also predicted for many other GT families [67]. The GT-A fold consists of an α/ß/α sandwich that resembles the Rossmann fold. Comparison of the catalytic domains of GT-A enzymes revealed the presence of two regions that are structurally well conserved [42]. The first region corresponds to the nucleotide binding domain (NBD, typically comprising around 100–120 residues) that is terminated by a characteristic Asp-Xxx-Asp sequence (also referred to as DxD motif). This motif is primarily involved in the binding of the phosphate groups of the nucleotide donor through the coordination of a divalent cation (*e.g.* Mn^2+^, Mg^2+^). The DxD motif can be easily identified since it is always located in a short loop connecting two ß-strands at the end of the NBD. The second conserved region (herein referred to as ß-α-α region) is located in the half C-terminal part of the catalytic domain of GT-A enzymes. It forms part of the active site and comprises residues that were shown to interact with both the donor and acceptor sugars [42]. In many inverting GT-A enzymes, the putative catalytic base (*i.e.* an aspartate or glutamate residue) was proposed in this region.

A GT-A fold could be predicted with high confidence (using the PHYRE programme) for four candidate sequences. The first one, At5g12260, is a predicted protein sequence of unknown function (no PFAM domain identified, Table S3) comprising 624 amino acids. Despite a high MR value with some of the selected guide genes, this unique gene in Arabidopsis could probably be related to cell wall synthesis as it is shown in ATTED-II database to be tightly coexpressed with several GT genes such as GAUT1 and GAUT7 that are listed in Table 1, and also with a putative UDP-GlcA/UDP-GalNAc transporter. Fold recognition analysis of At5g12260 protein sequence gives a very strong score for several GTs that adopt a GT-A fold, the highest score being obtained with the rabbit ß2-GlcNAc-Transferase I (GnT1, PDB code 1FO8). This allows to confidently assigning to this protein sequence a GT domain that encompasses residues ranging from [70 to ∼320]. At5g12260 displays a similar DxD motif as GnT1 (179-EDD) and comprises the structurally conserved ß- α-α region starting at position 235, with Asp263 that we propose as the putative catalytic residue. However examination of the C-terminal protein region [320–624] gave no indication for its function.

Two related protein sequences At1g61240 and At2g28310 are characterized by the presence of a DUF707 domain (also called PF05212) (Table S3). This domain is only present in proteins from plants and few bacteria. In *A. thaliana*, eleven related protein sequences with identities ranging from 33 to 84% exhibit this domain. At2g28310 has already been described as a putative GT [44]. PHYRE analysis only gave moderate scores with known GT-A folds, the best being the polypeptide-α-GalNAcT1 (PDB code 1XHB). However, using HCA, we were able to delineate the catalytic GT domain, starting at position around 115, to clearly identify a DxD motif (198-DED in At2g28310) and the conserved ß-α-α region in the C-terminal part of the catalytic domain. In At2g28310, this conserved region spans residues [265–305] with Asp298 being the probable catalytic residue.

One additional GT-A-like protein, At4g38500 known to localize in the Golgi apparatus [47] and containing a DUF616 domain was selected in the filtering methodology. Three other genes (At1g34550, At4g09630, At5g42660) encoding proteins containing this domain were initially pre-selected but finally filtered out since they were not significantly overexpressed under isoxaben treatment (Table S2). As a consequence, although these three genes do not seem to be involved in RG-II or pectin biosynthesis, they are likely to be linked to the primary cell wall formation. Despite high MR values with the Arabidopsis guide genes, At4g38500 is tightly co-expressed (MR<150) with other genes directly or indirectly related to pectin synthesis: Two GT genes (*QUA1/GAUT8* (Fig. 3) and *GAUT10*) and two genes involved in the metabolism of nucleotide sugars (*UXS1* and At5g15490 encoding respectively an UDP-GlcA decarboxylase and an UDP-Glc deshydrogenase). Similarly to the above candidates, At4g38500 protein sequence (499 aa) is predicted to share a GT-A fold. Using PHYRE, the best score was obtained for LgtC, a bacterial α4-GalT of family GT8 (PDB code 1GA8). HCA examination of the protein sequence allowed delineating the GT catalytic domain in the region [200–499], including a variant of the DxD motif (299-DGK) and the conserved ß-α-α region [380–420]. We failed to identify other conserved peptide motifs characteristics of GT8 family, such as the HxxGxxKPW motif [68]. This motif was shown in the crystal structure to lock the nucleotide sugar in the binding site [69]. The residue Asp409 in At4g38500 is proposed as the catalytic base. Despite a clear GT signature indicative of a GT-A fold, there is no way to assign a more precise biochemical function for these four protein sequences.

#### GT-B like sequences

All of the remaining non-CAZy candidate sequences listed in Table 1 are predicted to adopt a GT-B fold or variants of this fold that have been described for bacterial and eukaryotic fucosyltransferases belonging to GT10, GT23, GT65 and GT68 families [63],[70],[71],[72]. The canonical GT-B fold is characterized by two separate Rossmann-type domains with a connective linker region and a catalytic site located between the domains [42],[67]. In contrast to GT-A enzymes, GT-B enzymes are usually described as non-metal dependent. The human α6-FucT (hFUT8, GT23) has an unusual modular architecture consisting of a N-terminal coiled coil region, a catalytic domain and a C-terminal SH3 domain [72]. The catalytic domain is formed of two sub-structures, an open sheet α/ß structure and a classical Rossmann domain. The 3D structure of the catalytic domain of a protein-*O*-fucosyltransferase from *Caenorhabditis elegans* (POFUT1, GT65) and from human (POFUT2, GT68) have been very recently determined and they were shown to adopt the typical GT-B folding [63],[71].

**Figure 4:**
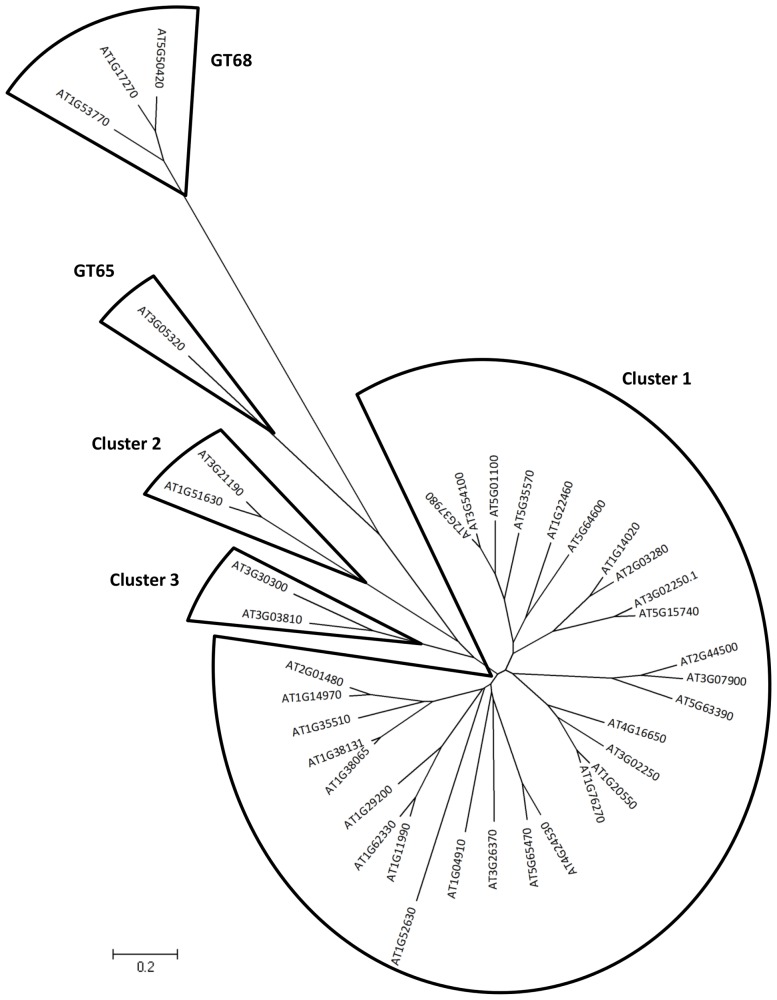
Phylogenetic tree of *Arabidopsis* sequences harboring a PF10250 (DUF246) domain.

**Figure 5:**
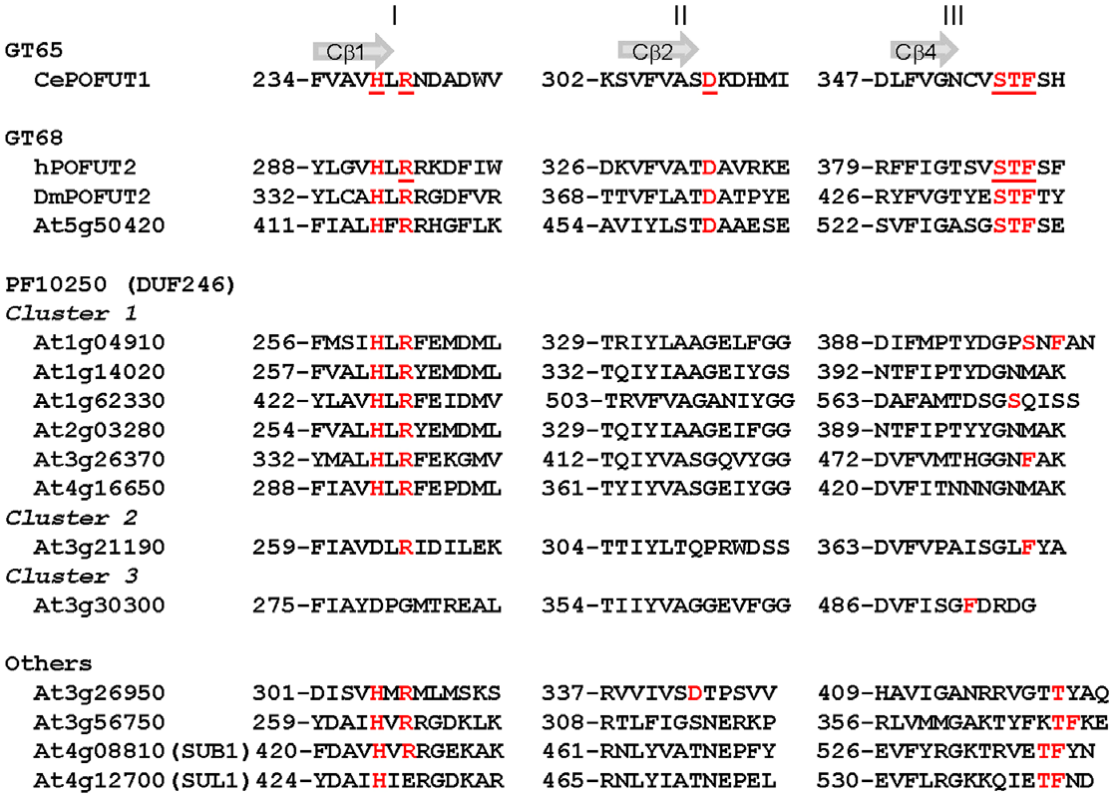
Multiple sequence alignement of GT-B like candidates (PF10250, DUF246). Multiple sequence alignments of GT-B like candidates with *C. elegans* POFUT1 and three GT68 fucosyltransferases, including the human POFUT2, showing the three conserved peptide regions (denoted I, II, and II). Amino acids indicated in red and underlined correspond to the catalytic HxR sequence in motif I, and D residue (motif II) and STF (motif III) that are involved in the binding of GDP [63],[71]. The accession numbers (from UniprotKB/Swiss-Prot) for the non plant sequences are: Q18014 (CePOFUT1), Q9W589 (DmPOFUT2) and Q9Y2G5 (hPOFUT2).

In our screening, eight protein sequences are annotated as containing a “protein-*O*-fucosyltransferase domain” (PFAM accession number PF10250, also called DUF246, Table S3). They are also annotated in the ARAMEMNON database as “putative Axi1-like membrane protein of unknown function” (Axi for auxin-independent growth promoter). Among these eight sequences, six sequences (At1g04910, At1g14020, At1g62330, At2g03280, At3g26370 and At4g16650) belong to the same protein cluster, that we will refer to as cluster 1, which comprises 30 Arabidopsis sequences displaying around 30% sequence identity with At1g04910 (Fig. 4). Although they are similarly annotated, the two other sequences, At3g21190 and At3g30300 are found in different clusters (clusters 2 and 3) (Fig. 4). Taken together, a total of 38 Arabidopsis sequences (and 27 *Oryza sativa* sequences) are annotated as “Axi1-like membrane protein” and/or as putative “protein *O*-fucosyltransferase” in ARAMEMNON. Only 4 out of the 38 sequences are currently classified in CAZy (in GT65 and GT68 families), the 34 remaining sequences are too distantly related to known GT functions to be included in CAZy. Only the use of remote homology detection methods such as PSI-BLAST [73] and Profile Hidden Markov Model (HMM) [74] allows retrieving of all sequences with a PF10250 signature. Why there are so many plant sequences with a PF10250 domain is a puzzling question. It must be stressed that protein members of families GT65 and GT68 belong to the large fucosyltransferase superfamily that has been previously described [64] and which also includes protein sequences from three other CAZy GT families (GT11, GT23, GT37). It is striking to note that all of the currently characterized biochemical functions in these GT families are fucosyltransferases (α2-FucTs, α6-FucTs and prot-*O*-FucTs). The known α3-FucTs and α4-FucTs classify in a different GT family (GT10) not included in the FucT superfamily. This superfamily is characterized by the presence of three conserved peptide regions (called motifs I, II, and III) [14],[64],[75] (Fig. 5). The recent crystal structures of *C. elegans* POFUT1 [71] and human POFUT2 [63], in complex with GDP-fucose, shed light on the function of these motifs. The three peptide motifs are located in the C-terminal domain that is mostly dedicated to the binding of the nucleotide sugar donor. Of particular interest is the motif I which comprises an invariant arginine (R240 in CePOFUT1) that was shown to be a key catalytic residue [71],[72],[75] (Fig. 5). This catalytic amino acid is located at the end of the first ß-strand of the C-terminal nucleotide binding domain and makes contact with the ß-phosphate (Fig. 5). Motif I also comprises a highly conserved His residue (H238) that was shown to interact with the guanine ring in *C. elegans* POFUT1 [71]. Motifs II and III are less conserved in the FucT superfamily [14]. They are also located at the end of ß-strands that form the central ß-sheet of the C-terminal domain. One acidic residue (D309) in motif II and a block of three residues (S355, T356, and F357) in motif III participate to the binding of GDP moiety. Fig. 5 shows the conservation of these motifs in POFUT1 and POFUT2 sequences and in plant sequences with a PF10250 (DUF246) domain. The HxR signature in motif I is well conserved in all plant sequences of cluster 1 except in At3g30300 sequence (cluster 3) which lacks this peptide signature and in At3g21190 (cluster 2) where only the arginine is conserved. None of the amino acids in motifs II and III that were shown in POFUT1 and POFUT2 structures to interact with GDP are strictly conserved in the non-CAZy plant sequences with a PF10250 domain.

Six of the 8 plant proteins harboring a PF10250 domain in Table 1 have been localized in the Golgi apparatus [47] (Table S4) suggesting a potential role in cell wall biosynthesis. Four genes also appear to be more promising candidates based on their MR values with guide genes and their own coexpression gene network (At1g04910, At1g62330, At3g26370 and At3g21190).

The four remaining GT-B-like protein sequences do not have a PF10250 domain, but similarly, they gave strong scores using PHYRE with FucT fold types, particularly with POFUT1 (PDB code 3ZY6) and NodZ (PDB code 2HLH) of families GT65 and GT23, respectively. At3g26950 is a unique gene in Arabidopsis which encodes a predicted 548-amino acid protein with no PFAM domain identified. It is tightly co-expressed with many other genes listed in Table 1 (*i.e.* At2g33570, At2g03280, At1g61240, At3g56750, At3g21190) and as such is a good candidate for RG-II synthesis. On the basis of fold recognition analysis, one can predict the occurrence of a GT domain in the region [120–450]. A similar observation could be made for At3g56750 (403 aa) with a GT domain predicted in the region [65–403] as regards fold prediction and FucT signature. At4g08810 (SUB1) and At4g12700 (SUL1) belong to the same gene cluster that comprises only 3 genes. SUB1 was previously proposed to be a calcium-binding protein involved in photomorphogenesis [76]. Present data suggest that SUB1 and related sequences (SUL1 and SUL2) could be GTs since PHYRE predicts with high confidence a GT domain in the half C-terminal of these proteins [250–550 in SUB1]. These four GT-B-like protein sequences exhibit the three conserved peptide regions, particularly the HxR signature in motif I (except in AtSUL1) (Fig. 5).

Altogether, the screening procedure identified 12 protein sequences for which a variant of the GT-B fold is predicted. This variant has only been observed in members of the FucT superfamily. Although it remains to be determined if the presence of the conserved peptide motifs depicted in Fig. 5, are always indicative of a FucT activity, the selected plant sequences could be considered as good candidates for the two fucosyltransferase activities that contribute to the synthesis of RG-II side chains. However, the absence of the HxR signature (motif I) in At3g30300 and At3g21190 may suggest a different glycosyltransferase activity for these two protein sequences.

## Discussion

In this study, we have set up a bioinformatics strategy aiming at identifying putative GTs involved in RG-II biosynthesis. This strategy is based on the selection of candidate genes encoding type II membrane proteins, the classical topology of Golgi GTs, and that are tightly coexpressed in both *Oryza sativa* and Arabidopsis with previously characterised genes encoding enzymes involved in the synthesis of RG-II. Among putative candidate genes, we conserved only those exhibiting an up-regulation in Arabidopsis upon isoxaben treatment because plants submitted to this herbicide compensate the decrease of cellulose by accumulating HA [44]. The biosynthesis of xyloglucan and pectins are closely related to ensure an appropriate deposition of polysaccharides within primary cell walls. Although they exhibit coexpression patterns similar to those of query genes, genes encoding a putative RG-I GT (ARAD1) or xyloglucan-specific GTs (FUT1, MUR3) are not over-expressed upon isoxaben treatment which confirms that the herbicide mainly induces the overexpression of genes involved in HA synthesis (Table S2). We postulate that genes encoding RG-II GTs are also overexpressed under isoxaben treatment because this pectic molecule is composed of HA substituted by four side-chains. It is worth noting that the overexpression of Arabidopsis guide genes, including AtRGXT4, in isoxaben-habituated cells supports this hypothesis [44]. However, we cannot definitively rule out that some of the candidate GTs selected in this study are not related to the biosynthesis of pectins but to other polysaccharides or glycoconjugates of which synthesis is tightly coexpressed with HA. Similarly, one cannot exclude that the filter strategy used also led to the removal of potential RG-II relevant genes at intermediate steps. This is the reason why we provided the list of rejected candidates in Tables S1 and S2.

This study results in the final selection of 26 Arabidopsis gene families encoding putative GTs, including 10 CAZy GTs. Among the CAZy sequences, we selected 4 four potential α-galacturonosyltransferase candidates, three of them being involved in the synthesis of α4-GalA oligogalacturonides constitutive of both HA and RG-II, as well as two sialyltransferases-like (ST-like) previously proposed to be involved in the transfer of Kdo and/or Dha on the pectic backbone of RG-II [58]. Because there are many examples of closely related sequences having different catalytic activity and also because most of the CAZy families are polyspecific, it is extremely difficult to predict the biochemical function of other putative GTs on the basis of sequence similarity [42],[67]. Putative functions have been proposed in this study for GAUT4, GT31 and GT92 candidates but these must be experimentally confirmed either through the study of defective mutants or biochemical transferase assays. This is even truer for the 16 non-CAZy GT sequences that were retrieved in the present study. Twelve of these sequences exhibit a FucT signature and therefore they are good candidates for the two FucT activities that are required for RG-II synthesis. However, given the high number of Arabidopsis sequences harbouring this FucT signature (∼38 PF10250-annotated genes), it is thus questionable if the presence of this peptide signature is always indicative of a FucT activity. One cannot exclude other GT functions for these sequences and a plausible assumption would be enzymes using a different GDP-sugar, such as the GDP-ß-L-Gal required for transfer of the terminal L-Gal residue on the side chain A (Fig. 1). It should also be considered that these protein sequences are involved in the transfer of rare monosaccharides (*i.e.* AceA) about which nothing is known of their activated forms in the plant cell.

The analysis of Arabidopsis mutants altered in candidate genes selected in this study may answer about their effective involvement in RG-II biosynthesis. Mutation in one of the two GT29 genes (At1g08660) was demonstrated to induce defects in pollen germination and pollen tube growth [59]. Interestingly, efforts to select Arabidopsis homozygous mutant lines for the second sequence (At3g48820) were unsuccessful (unpublished data) suggesting that this sequence is also involved in pollen development as reported for mutants impaired in Kdo synthesis [10]. Similarly, the lack of recoverable mutants for GAUT4 highlights the importance of this gene in plant growth and development [55]. Among members of the FucT superfamily selected in this study, the biological function of At4g08810 (SUB1) has been previously investigated. The *sub1* (short under blue light) mutant was demonstrated to exhibit a short hypocotyl growth both in blue light (BL) and far-red light (FRL) of relatively low fluence rates (<10 µmol.m^−2^.s^−1^) and a high expression level of genes encoding flavonoid biosynthetic enzymes [76]. SUB1 was in consequence proposed to be a calcium-binding protein playing a role in photomorphogenetic responses. In contrast, biological function of SUL1 has actually not been investigated. For other candidate genes, no information is available so far with regards to the phenotype of the corresponding mutants.

The bioinformatics strategy used in the present study allowed retrieval of 26 orthologous gene pairs potentially encoding GT candidates for RG-II synthesis. Protein sequence analysis has enabled us to hypothesize a biochemical function for some of these genes. One challenging task will be the experimental validation to obtain direct evidence of their involvement in RG-II synthesis. This could be achieved through the analysis of mutant wall composition phenotypes or the demonstration of a glycosyltransferase activity on appropriate substrates.

## Supporting Information

Figure S1 **Phylogenetic tree of GT4 rice and** ***A. thaliana*** **sequences.** Putative RG-II GTs selected in this study are circled. At : *Arabidopsis thaliana*, Os : *Oryza sativa*.
(TIF)

Figure S2 **CAZy GT68 phylogenetic tree.** Plant specific clade is circled. Ag: *Anopheles gambiae*, Ap: *Acyrthosiphon pisum*, As: *Ascaris suum*, At : *Arabidopsis thaliana*, Bm: *Bombyx mori*, Bt: *Bos taurus*, Cb: *Caenorhabditis briggsae*, Ce: *Caenorhabditis elegans*, Ci: *Ciona intestinalis*, Cs: *Ciona savignyi*, Dm: *Drosophila melanogaster*, Dp: *Drosophila pseudoobscura*, Dr : *Danio rerio, Dy: Drosophila yakuba*, Gg: *Gallus gallus*, Gm: *Glycine max*, Ha: *Helianthus annuus*, Hs: *Homo sapiens*, Mm: *Mus musculus*, Nc: *Neospora caninum Liverpool*, Ol : *Oryzias latipes*, Os : *Oryza sativa*, Pf: *Plasmodium falciparum 3D7*, Pk: *Plasmodium knowlesi strain H*, Pt: *Pan troglodytes*, Py: *Plasmodium yoelii*, Sm: *Schistosoma mansoni*, St: *Solanum tuberosum*, Tr: *Takifugu rubripes*, Vv: *Vitis vinifera*, Xt: *Xenopus (Silurana) tropicalis*. The underlined Dm-AAK77300 sequence corresponds to POFUT2 transferase characterized in Drosophila [62].
(TIF)

Figure S3 **CAZy GT92 phylogenetic tree.** Plant specific clade is circled. Aa: *Aedes aegypti*, Ag: *Anopheles gambiae str. PEST*, As: *Ascaris suum*, At : *Arabidopsis thaliana*, Bm: *Brugia malayi*, Cb: *Caenorhabditis briggsae AF16*, Ce: *Caenorhabditis elegans*, Ch: *Cryptosporidium hominis*, Cl: *Columba livia*, Cm: *Cryptosporidium muris RN66*, Cp: *Cryptosporidium parvum Iowa II*, Da: *Drosophila ananassae*, De: *Drosophila erecta*, Dg: *Drosophila grimshawi*, Dm: *Drosophila melanogaster*, Dp: *Drosophila persimilis*, Dpp: *Drosophila pseudoobscura pseudoobscura*, Dr: *Danio rerio*, Ds: *Drosophila sechellia*, Dv: *Drosophila virilis*, Dw: *Drosophila willistoni*, Dy: *Drosophila yakuba*, Hv: *Hordeum vulgare subsp. vulgare*, Is: *Ixodes scapularis*, Nv: *Nematostella vectensis*, Os : *Oryza sativa*,Pp: *Physcomitrella patens subsp. Patens*, Pt: *Populus trichocarpa*, Rc: *Ricinus communis*, Tn: *Tetraodon nigroviridis*, Vv: *Vitis vinifera*, Xl: *Xenopus laevis*, Xt: *Xenopus (Silurana) tropicalis*, Zm: *Zea mays*.
(TIF)

Table S1 **List of genes from both Arabidopsis and rice that have been rejected in the phylogenetic profiling screening step (filter IV).**
(DOC)

Table S2 **List of genes that have been rejected in the isoxaben screening step (filter VI).** Analysis of microarray data obtained from isoxaben-habituated Arabidopsis indicated that, except for XXT2 (see the text), these genes are not overexpressed upon isoxaben treatment [44].
(DOC)

Table S3 **Sequence information regarding CAZy and non-CAZy GT candidates selected in this study.** A) Length and location of the transmembrane domain (TMD) of CAZy GTs. B) Length, location of TMD and domains (PFAM domains and/or GT domains identified using HCA), and highest score obtained using PHYRE. ^a^ PDB code according to PHYRE results.
(DOC)

Table S4 **Golgi localisation according to Parsons et al.** [**47**] **.** *AtSUL2 homologue was identified in the Golgi proteome.
(DOC)
