# Supplementary figures and images for: Identification of Putative Rhamnogalacturonan-II Specific Glycosyltransferases in Arabidopsis Using a Combination of Bioinformatics Approaches
Source: PLoS One. 2012 Dec 14;7(12):e51129. doi: 10.1371/journal.pone.0051129 (PMC3522684; doi:10.1371/journal.pone.0051129)

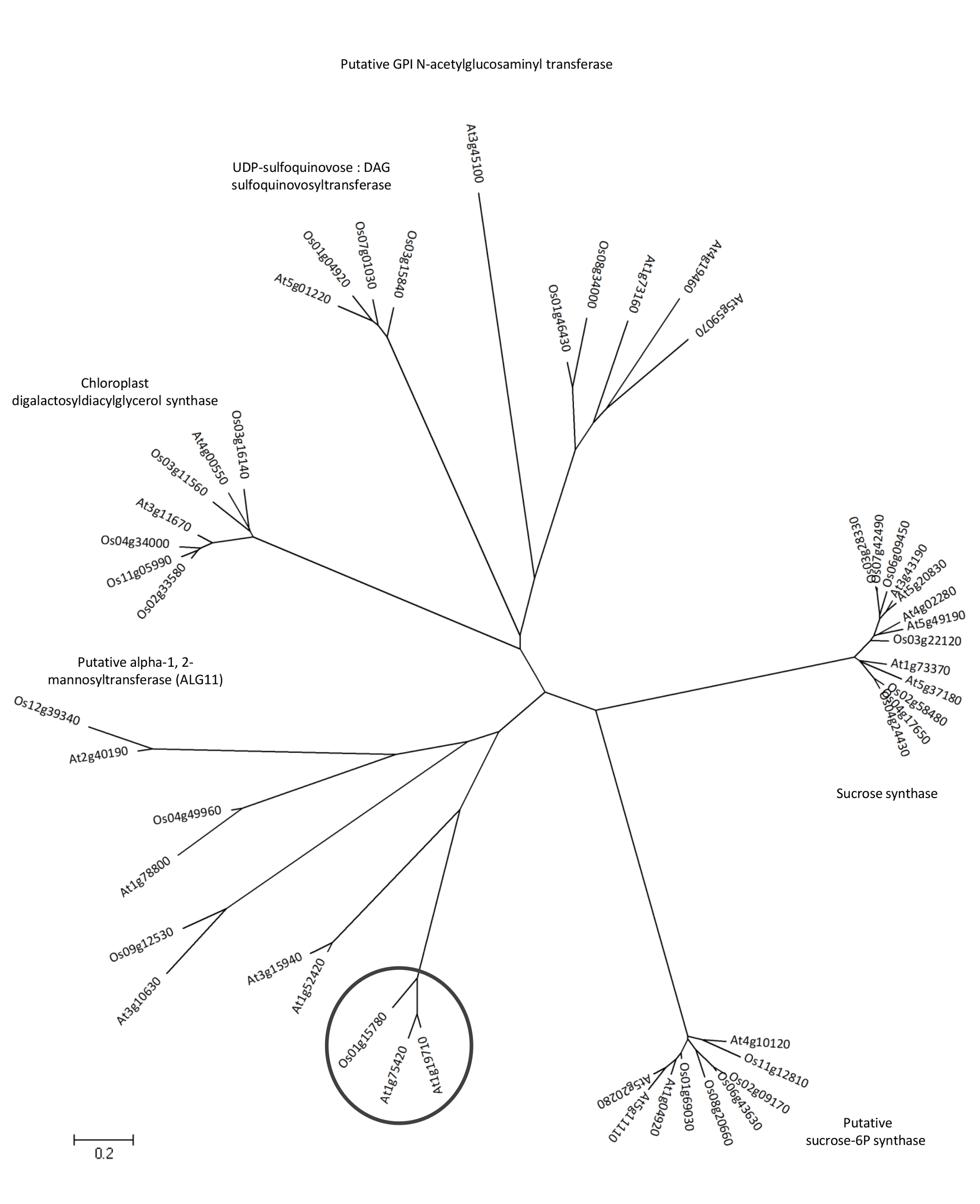

Supplement: Figure S1 — Phylogenetic tree of GT4 rice and A. thaliana sequences. Putative RG-II GTs selected in this study are circled. At : Arabidopsis thaliana, Os : Oryza sativa. (TIF) [file pone.0051129.s001.tif]

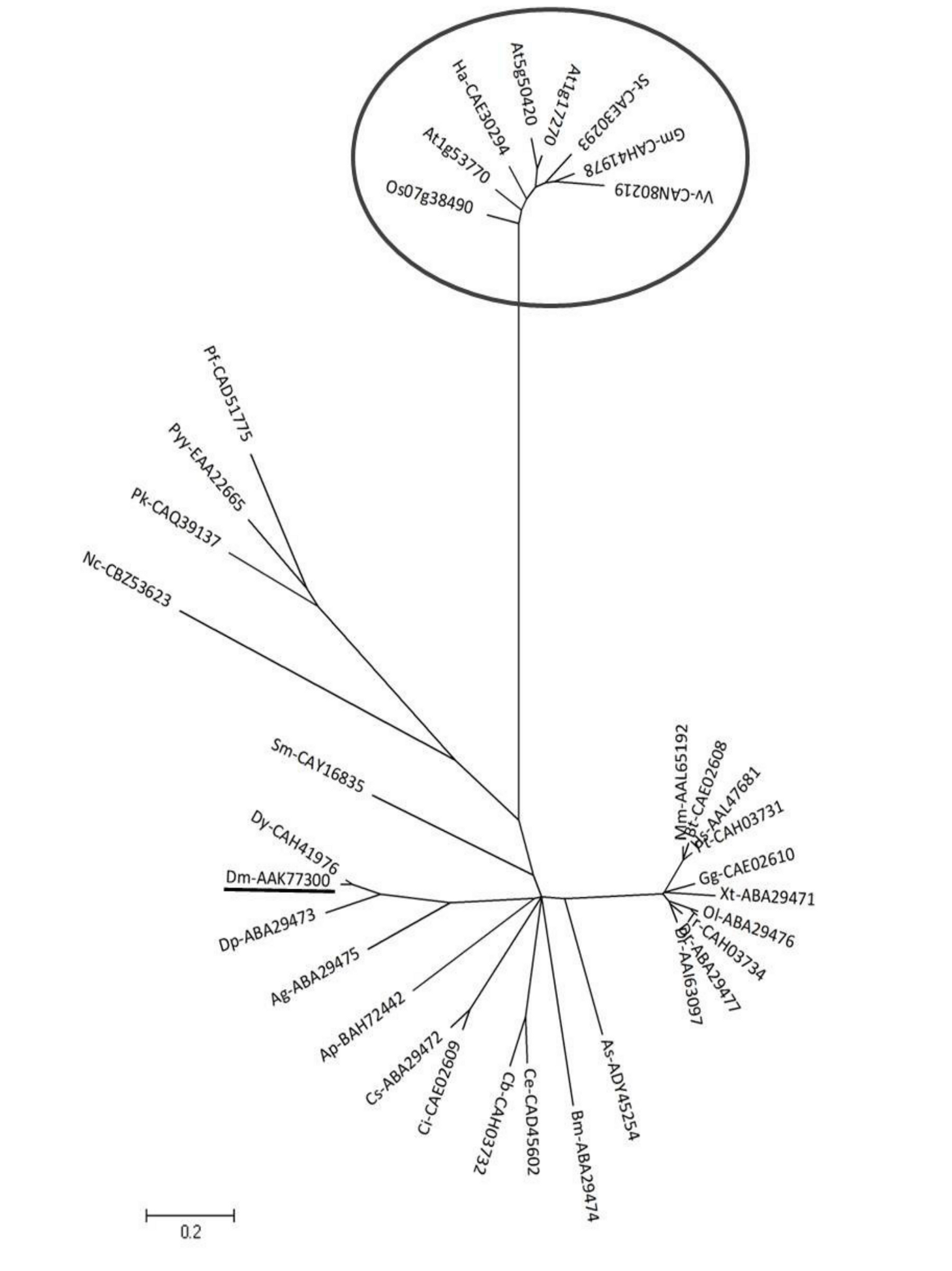

Supplement: Figure S2 — CAZy GT68 phylogenetic tree. Plant specific clade is circled. Ag: Anopheles gambiae, Ap: Acyrthosiphon pisum, As: Ascaris suum, At : Arabidopsis thaliana, Bm: Bombyx mori, Bt: Bos taurus, Cb: Caenorhabditis briggsae, Ce: Caenorhabditis elegans, Ci: Ciona intestinalis, Cs: Ciona savignyi, Dm: Drosophila melanogaster, Dp: Drosophila pseudoobscura, Dr : Danio rerio, Dy: Drosophila yakuba, Gg: Gallus gallus, Gm: Glycine max, Ha: Helianthus annuus, Hs: Homo sapiens, Mm: Mus musculus, Nc: Neospora caninum Liverpool, Ol : Oryzias latipes, Os : Oryza sativa, Pf: Plasmodium falciparum 3D7, Pk: Plasmodium knowlesi strain H, Pt: Pan troglodytes, Py: Plasmodium yoelii, Sm: Schistosoma mansoni, St: Solanum tuberosum, Tr: Takifugu rubripes, Vv: Vitis vinifera, Xt: Xenopus (Silurana) tropicalis. The underlined Dm-AAK77300 sequence corresponds to POFUT2 transferase characterized in Drosophila [62]. (TIF) [file pone.0051129.s002.tif]

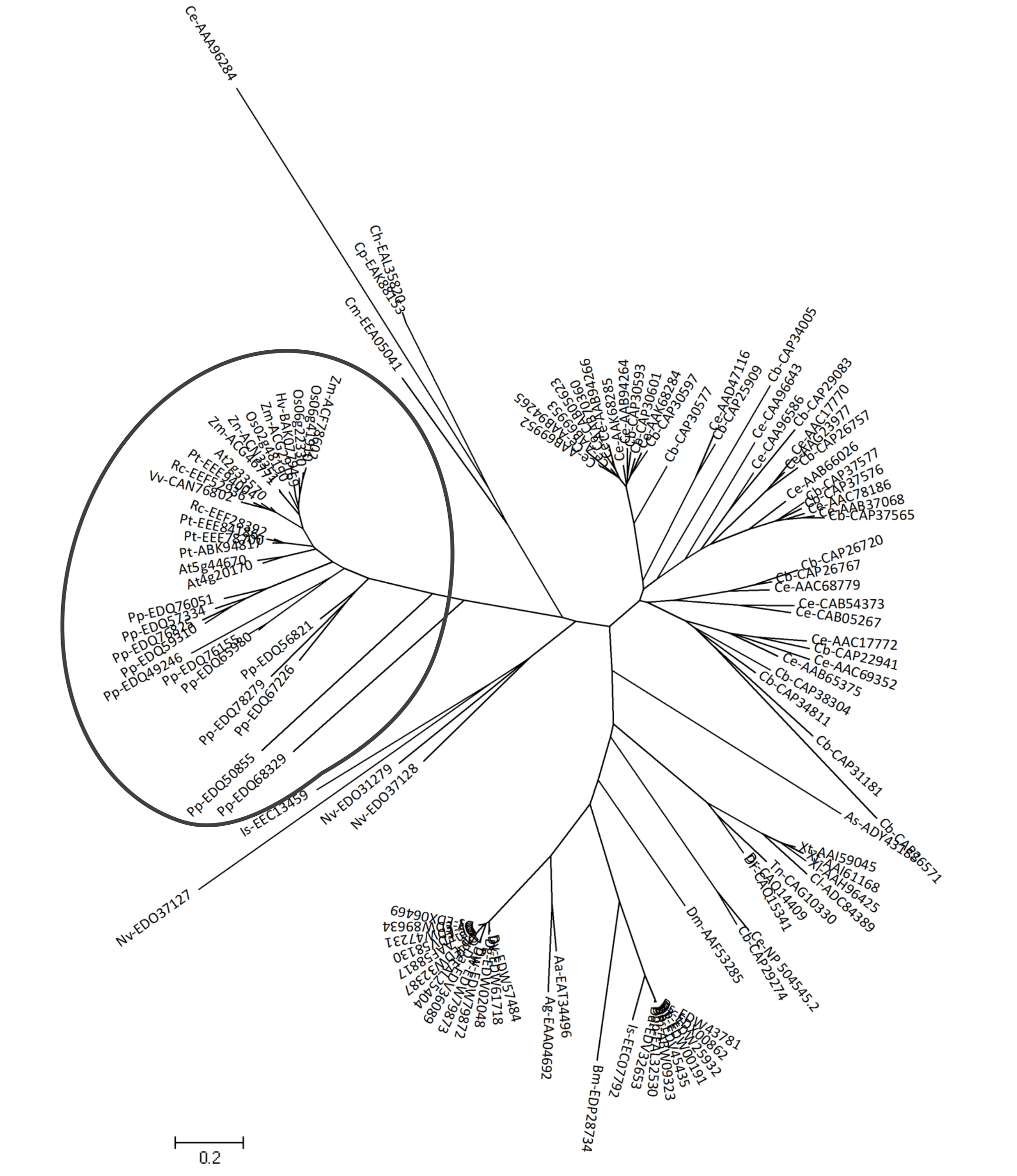

Supplement: Figure S3 — CAZy GT92 phylogenetic tree. Plant specific clade is circled. Aa: Aedes aegypti, Ag: Anopheles gambiae str. PEST, As: Ascaris suum, At : Arabidopsis thaliana, Bm: Brugia malayi, Cb: Caenorhabditis briggsae AF16, Ce: Caenorhabditis elegans, Ch: Cryptosporidium hominis, Cl: Columba livia, Cm: Cryptosporidium muris RN66, Cp: Cryptosporidium parvum Iowa II, Da: Drosophila ananassae, De: Drosophila erecta, Dg: Drosophila grimshawi, Dm: Drosophila melanogaster, Dp: Drosophila persimilis, Dpp: Drosophila pseudoobscura pseudoobscura, Dr: Danio rerio, Ds: Drosophila sechellia, Dv: Drosophila virilis, Dw: Drosophila willistoni, Dy: Drosophila yakuba, Hv: Hordeum vulgare subsp. vulgare, Is: Ixodes scapularis, Nv: Nematostella vectensis, Os : Oryza sativa,Pp: Physcomitrella patens subsp. Patens, Pt: Populus trichocarpa, Rc: Ricinus communis, Tn: Tetraodon nigroviridis, Vv: Vitis vinifera, Xl: Xenopus laevis, Xt: Xenopus (Silurana) tropicalis, Zm: Zea mays. (TIF) [file pone.0051129.s003.tif]
