# Supplementary material for: Identification of Putative Rhamnogalacturonan-II Specific Glycosyltransferases in Arabidopsis Using a Combination of Bioinformatics Approaches
Source: PLoS One. 2012 Dec 14;7(12):e51129. doi: 10.1371/journal.pone.0051129 (PMC3522684; doi:10.1371/journal.pone.0051129)
Supplement: Table S1 — List of genes from both Arabidopsis and rice that have been rejected in the phylogenetic profiling screening step (filter IV). (DOC) [file pone.0051129.s004.doc]

**Supplemental Table S1.** *List of genes from both Arabidopsis and rice that have been rejected in the phylogenetic profiling screening step (filter IV).*

| Locus Identifier | Gene description |
| --- | --- |
| At1g13250 | GALACTURONOSYLTRANSFERASE-LIKE 3 (GATL3) |
| At1g16570 | GT33 |
| At1g53290 | GT31 |
| At1g71990 | Encodes a Lewis-type alpha 1,4-fucosyltransferase (FUCTC) |
| At2g26680 | unknown protein |
| At2g41770 | DUF288 |
| At2g42570 | DUF231 |
| At3g06060 | Encodes one of the Arabidopsis proteins (At3g06060/TSC10A and At5g19200/TSC10B) with significant similarity to the yeast 3-ketodihydrosphinganine (3-KDS) reductase, Tsc10p |
| At3g06440 | GT31 |
| At3g10630 | GT4 |
| At3g15940 | GT4 |
| At3g57420 | DUF288 |
| At4g02130 | GALACTURONOSYLTRANSFERASE 6 (GATL6) |
| At4g11090 | DUF231 |
| At4g15820 | unknown protein |
| At4g19900 | GT32 |
| At4g22580 | Exostosin family protein |
| At4g26130 | unknown protein |
| At4g32120 | GT31 |
| At5g01620 | DUF231 |
| At5g16720 | DUF593 |
| At5g19670 | Exostosin family protein (GT47) |
| At5g65650 | DUF1195 |
| Os01g10490 | unknown protein |
| Os01g42810 | DUF231 |
| Os02g10190 | DUF1680 |
| Os03g21090 | O-fucosyltransferase family protein |
| Os03g24300 | Unknown |
| Os03g38930 | Nucleotide-diphospho-sugar transferase |
| Os03g58490 | DUF593 |
| Os03g60340 | DUF231 |
| Os04g49140 | DUF1191 |
| Os05g11060 | putative galactosyltransferase |
| Os05g48600 | putative class GT43 beta-glucuronyltransferase |
| Os06g46570 | putative galactosyltransferase |
| Os07g49370 | putative class GT43 beta-glucuronyltransferase |
| Os08g02370 | putative galactosyltransferase |
| Os09g23360 | DUF231 |
| Os11g03160 | putative galacturonosyltransferase |
| Os11g48050 | DUF740 |
| Os12g01560 | DUF231 |
