# Supplementary material for: Identification of Putative Rhamnogalacturonan-II Specific Glycosyltransferases in Arabidopsis Using a Combination of Bioinformatics Approaches
Source: PLoS One. 2012 Dec 14;7(12):e51129. doi: 10.1371/journal.pone.0051129 (PMC3522684; doi:10.1371/journal.pone.0051129)
Supplement: Table S2 — List of genes that have been rejected in the isoxaben screening step (filter VI). Analysis of microarray data obtained from isoxaben-habituated Arabidopsis indicated that, except for XXT2 (see the text), these genes are not overexpressed upon isoxaben treatment [44]. (DOC) [file pone.0051129.s005.doc]

**Supplemental Table S2.** *List of genes that have been rejected in the isoxaben screening step (filter VI). Analysis of microarray data obtained from isoxaben-habituated Arabidopsis indicated that, except for XXT2 (see the text), these genes are not overexpressed upon isoxaben treatment* ***[44]****.*

| **Locus** | **Gene description** |
| --- | --- |
| At3g02250 | O-fucosyltransferase family protein |
| At5g35570 | O-fucosyltransferase family protein |
| At1g34270 | Exostosin family protein GT47 |
| At4g38040 | Exostosin family protein GT47 |
| At4g22580 | Exostosin family protein GT47 |
| At2g35100 | ARABINAN DEFICIENT 1 (ARAD1) |
| At2g20810 | GAUT10 |
| At1g18580 | GAUT11 |
| At3g02350 | GAUT7 |
| At2g20370 | MUR3 |
| At1g74380 | XXT5 |
| At4g02500 | XXT2 |
| At2g03220 | FUT1 |
| At5g42660 | DUF616 |
| At4g09630 | DUF616 |
| At1g34550 | DUF616, EMBRYO DEFECTIVE 2756 (EMB2756) |
