# Supplementary material for: Identification of Putative Rhamnogalacturonan-II Specific Glycosyltransferases in Arabidopsis Using a Combination of Bioinformatics Approaches
Source: PLoS One. 2012 Dec 14;7(12):e51129. doi: 10.1371/journal.pone.0051129 (PMC3522684; doi:10.1371/journal.pone.0051129)
Supplement: Table S3 — Sequence information regarding CAZy and non-CAZy GT candidates selected in this study. A) Length and location of the transmembrane domain (TMD) of CAZy GTs. B) Length, location of TMD and domains (PFAM domains and/or GT domains identified using HCA), and highest score obtained using PHYRE. a PDB code according to PHYRE results. (DOC) [file pone.0051129.s006.doc]

**Supplemental Table S3.** *Sequence information regarding* *CAZy and non-CAZy GT* candidates selected in this study. A) Length and location of the transmembrane domain *(TMD) of CAZy GTs. B) Length, location of TMD and domains (PFAM domains and/or GT domains identified using HCA), and highest score obtained using PHYRE. ^a^ PDB code according to PHYRE results.*

| **A.** |  | | **Length (aa)** | | **TMD** |
| --- | --- | --- | --- | --- | --- |
| **CAZy GT** |  |  | |  | |
| **GT4** |  |  | |  | |
| At1g19710 |  | 479 | | 16-36 | |
| **GT8** |  |  | |  | |
| At5g47780 | **AtGAUT4** | 616 | | 5-25 | |
| At2g38650 | **AtGAUT7** | 619 | | 21-41 | |
| At3g25140 | **AtGAUT8** | 559 | | 19-39 | |
| At3g61130 | **AtGAUT1** | 673 | | 21-41 | |
| **GT29** |  |  | |  | |
| At1g08660 |  | 474 | | 10-30 | |
| At3g48820 |  | 440 | | 4-24 | |
| **GT31** |  |  | |  | |
| At5g53340 |  | 338 | | 13-33 | |
| **GT68** |  |  | |  | |
| At5g50420 |  | 566 | | 63-83 | |
| **GT92** |  |  | |  | |
| At2g33570 |  | 496 | | 19-39 | |

| **B.** |  | |  |  | **Pfam** | |  | **GT domain** | |
| --- | --- | --- | --- | --- | --- | --- | --- | --- | --- |
|  |  | | **Length (aa)** | **TMD** | **Start-end** | **E-value** |  | **Start-end** | **Top GT template^a^** |
| **Non CAZy GT** | | |  |  |  |  |  |  |  |
| **GT-A like** | |  |  |  |  |  |  |  |  |
| **No PFAM** | |  |  |  |  |  |  |  |  |
| At5g12260 | |  | 624 | 25-45 |  | - |  | 70-320 | 1FO8 |
| **DUF616** | |  |  |  |  |  |  |  |  |
| At4g38500 | |  | 499 | 54-74 | 138-443 | 4.2e-134 |  | 200-499 | 1GA8 |
| **DUF707 (PF05212)** | |  |  |  |  |  |  |  |  |
| At1g61240 | |  | 425 | 29-49 | 81-384 | 4.1e-118 |  | 117-320 | 3BCV |
| At2g28310 | |  | 374 | 18-38 | 76-367 | 2.3e-162 |  | 115-329 | 1XHB |
| **GT-B like** | |  |  |  |  |  |  |  |  |
| **DUF246 (PF10250)** | |  |  |  |  |  |  |  |  |
| At1g04910 | |  | 519 | 22-42 | 95-414 | 1.2e-83 |  | 87-415 | 3ZY6 |
| At1g14020 | |  | 499 | 30-50 | 97-416 | 1.6e-91 |  | 91-417 | 3ZY6 |
| At2g03280 | |  | 481 | 32-52 | 164-441 | 4.5e-69 |  | 88-414 | 3ZY6 |
| At4g16650 | |  | 549 | 44-64 | 130-444 | 6.2e-93 |  | 122-445 | 3ZY6 |
| At1g62330 | |  | 652 | 89-109 | 227-591 | 2.4e-92 |  | 221-592 | 3ZY6 |
| At3g26370 | |  | 557 | 79-99 | 166-498 | 4.5e-83 |  | 157-499 | 3ZY6 |
| At3g30300 | |  | 677 | 13-33 | 108-439 | 2.6e-64 |  | 100-519 | 3ZY6 |
| At3g21190 | |  | 422 | 3-23 | 108-388 | 8.7e-23 |  | 87-388 | 3ZY6 |
| **Other** | |  |  |  |  |  |  |  |  |
| At3g26950 | |  | 548 | 12-32 | - | - |  | 120-450 | 2HLH |
| At3g56750 | |  | 403 | 17-37 | - | - |  | 65-403 | 3ZY6 |
| At4g12700 | | SUL1 | 561 | 24-44 | - | - |  | 254-541 | 3ZY6 |
| At4g08810 | | SUB1 | 552 | 18-38 | - | - |  | 250-550 | 3ZY6 |
