# Supplementary material for: Identification of Putative Rhamnogalacturonan-II Specific Glycosyltransferases in Arabidopsis Using a Combination of Bioinformatics Approaches
Source: PLoS One. 2012 Dec 14;7(12):e51129. doi: 10.1371/journal.pone.0051129 (PMC3522684; doi:10.1371/journal.pone.0051129)
Supplement: Table S4 — Golgi localisation according to Parsons et al. [47] . *AtSUL2 homologue was identified in the Golgi proteome. (DOC) [file pone.0051129.s007.doc]

**Supplemental Table S4.** *Golgi localisation according to Parsons et al.* ***[47]****. *AtSUL2 homologue was identified in the Golgi proteome.*

|  | **Golgi**  **Localisation** |  | **Golgi**  **localisation** |
| --- | --- | --- | --- |
| **CAZy GT** |  | **Non CAZy GT** |  |
| **GT4** |  | **GT-A like** |  |
| At1g19710 | Y | No PFAM |  |
| **GT8** |  | At5g12260 | Y |
| At5g47780 | Y | DUF616 |  |
| At2g38650 | Y | At4g38500 | Y |
| At3g25140 | Y | DUF707 (PF05212) |  |
| At3g61130 | Y | At1g61240 | - |
| **GT29** |  | At2g28310 | Y |
| At1g08660 | Y | **GT-B like** |  |
| At3g48820 | Y | **DUF246 (PF10250)** |  |
| **GT31** |  | At1g04910 | Y |
| At5g53340 | Y | At1g14020 | - |
| **GT68** |  | At2g03280 | - |
| At5g50420 | Y | At4g16650 | Y |
| **GT92** |  | At1g62330 | Y |
| At2g33570 | - | At3g26370 | Y |
|  |  | At3g30300 | Y |
|  |  | At3g21190 | Y |
|  |  | **Other** |  |
|  |  | At3g26950 | - |
|  |  | At3g56750 | Y |
|  |  | At4g12700 | -* |
|  |  | At4g08810 | - |
